# A cluster-randomized, controlled trial of nutritional supplementation and promotion of responsive parenting in Madagascar: the MAHAY study design and rationale

**DOI:** 10.1186/s12889-016-3097-7

**Published:** 2016-06-03

**Authors:** Lia C. H. Fernald, Emanuela Galasso, Jumana Qamruddin, Christian Ranaivoson, Lisy Ratsifandrihamanana, Christine P. Stewart, Ann M. Weber

**Affiliations:** School of Public Health, University of California, Berkeley, 50 University Hall, MC 7360, Berkeley, CA 94720-7360 USA; Development Research Group, The World Bank, Washington D.C., USA; Health, Nutrition and Population Global Practice, The World Bank, Washington D.C., USA; Programme National de la Nutrition Communautaire, Antananarivo, Madagascar; Centre Médico-Educatif ‘Les Orchidées Blanches’, Antananarivo, Madagascar; Program in International and Community Nutrition, University of California, Davis, Davis, CA USA; School of Medicine, Stanford University, Stanford, CA USA

## Abstract

**Background:**

Over half of the world’s children suffer from poor nutrition, and as a consequence they experience delays in physical and mental health, and cognitive development. There is little data evaluating the effects of delivery of lipid-based, nutrition supplementation on growth and development during pregnancy and early childhood within the context of a scaled-up program. Furthermore, there is limited evidence on effects of scaled-up, home-visiting programs that focus on the promotion of child development within the context of an existing, national nutrition program.

**Methods/Design:**

The MAHAY ("smart" in Malagasy) study uses a multi-arm randomized-controlled trial (RCT) to test the effects and cost-effectiveness of combined interventions to address chronic malnutrition and poor child development. The arms of the trial are: (T_0_) existing program with monthly growth monitoring and nutritional/hygiene education; (T_1_) is T_0_ + home visits for intensive nutrition counseling within a behavior change framework; (T_2_) is T_1_ + lipid-based supplementation (LNS) for children 6–18 months old; (T_3_) is T_2_ + LNS supplementation of pregnant/lactating women; and (T_4_) is T_1_ + intensive home visiting program to support child development. There are anticipated to be *n* = 25 communities in each arm (*n* = 1250 pregnant women, *n* = 1250 children 0–6 months old, and *n* = 1250 children 6–18 months old). Primary outcomes include growth (length/height-for-age z-scores) and child development (mental, motor and social development). Secondary outcomes include care-giver reported child morbidity, household food security and diet diversity, micro-nutrient status, maternal knowledge of child care and feeding practices, and home stimulation practices. We will estimate unadjusted and adjusted intention-to-treat effects.

Study protocols have been reviewed and approved by the Malagasy Ethics Committee at the Ministry of Health in Madagascar and by the institutional review board at the University of California, Davis. This study is funded by the Strategic Impact Evaluation Fund (SIEF), the World Bank Innovation Grant, the Early Learning Partnership Grant, the Japan Scaling-up for Nutrition Trustfund, and Grand Challenges Canada. The implementation of the study is financed by Madagascar's National Nutrition Office.

**Trial registration:**

Current Controlled Trials ISRCTN14393738. Registered June 23, 2015.

## Background

### Rationale and background for the study

Hundreds of millions of the world’s children suffer from poor nutrition, and as a consequence they experience delays in physical and mental health and cognitive development [1]. Linear growth retardation (stunting) is associated with poor cognitive, language and behavioral development [2], high rates of morbidity and mortality, and negative long term outcomes including compromised labor market participation [3, 4]. Outcomes are even worse for severely stunted children [3, 5–7]. Despite evidence of the potential effectiveness of interventions in early childhood, policy makers and planners are challenged by designing interventions that sustainably improve growth and child development, particularly in the context of extreme poverty. Furthermore, since linear growth retardation is largely irreversible after the age of two or three [8], there is an urgent need to design interventions that begin early in life.

Madagascar is a very low-income country (gross national annual income per capita of 430 USD [9]), with a prevalence of stunting of almost 50 % [1]. Children in Madagascar living in poverty show deficits in developmental outcomes, such as language and cognition [10]. In order to address the severity and prevalence of poor nutrition in Madagascar, the National Office of Nutrition (ONN) initiated a large-scale community-based nutrition intervention in 1999, which is still operational throughout the country. The community-based program includes monthly growth-monitoring activities for infants and young children, cooking demonstrations, community mobilization, and nutrition and hygiene education of their primary caregivers. A rigorous multi-year evaluation of the long term effects of the existing program on weight-for-age showed small, significant effects of the program for young children, but no effects of the program on height-for-age [11]. To address the lack of effectiveness of Madagascar’s current program on linear growth faltering, the interventions described here were developed in collaboration with the research team and Madagascar’s Office of Nutrition.

The proposed evaluation has as its goal to identify the most effective, cost-effective and scalable interventions to address linear growth faltering and promote child development in Madagascar. Our study uses a multi-arm randomized-controlled trial (RCT) to test the effects and cost-effectiveness of interventions designed to address chronic malnutrition and poor child development, and has been designed based on the existing evidence [3, 12, 13]. The questions we are addressing through this research are described in detail below.

### Question 1: How do intensive counseling and/or nutritional supplementation affect linear growth faltering and child development?

Common strategies to improve nutritional status include the promotion of behavior change to increase dietary quality; the fortification of food to improve micronutrient content of staple foods; or the use of nutritional supplements for vulnerable population groups (e.g. very young children) [13–15]. Our study aims to test the added value of variations of these strategies over and above the existing, community-based, growth monitoring. Growth monitoring and education strategies are only successful when they involve intensive counseling and strongly emphasize dietary diversity and the promotion and consumption of animal source foods; [14] the quality of counseling and its intensity are believed to be a necessary condition for growth monitoring programs to be effective [16]. Two existing programs (one in Peru [17], and the other in China [18]) have demonstrated that primary caregivers are able to act on the advice of community nutrition workers to provide higher quality, micronutrient rich foods. Similarly, home visits in Malawi including nutritional information from birth to the first year of life showed significant improvements in the consumption of protein rich foods and in measures of height for age [19]. The findings are in line with evidence from behavioral economics on the importance of the way the messages are framed, perceived and internalized [20].

In spite of these encouraging studies, intensive counseling and behavioral change may not be sufficient to address severe nutritional deficiencies in settings with high food insecurity and intense poverty. Thus, nutritional supplementation may be a more effective option and can come in the form of micronutrient tablets or drops, multiple micronutrient powders (e.g. “Sprinkles”) mixed into the child’s typical complementary foods, or lipid-based nutrient supplements (LNS). While multiple micronutrient powders appear to be effective for improving hemoglobin, iron status and some cognitive outcomes in children, they have shown limited effects on child growth [21, 22]. Multiple micronutrient supplements in pregnancy have been found to have a small impact on birth weight [23], though the effects appear to be minimal in women of low BMI in early pregnancy. For LNS findings, evidence is emerging from a number of efficacy trials in Africa and Asia that suggest there are important benefits of LNS products to infant growth and development [24]. For example, in Malawi, LNS supplementation reduced severe stunting to 3.5 % or less as compared to 12.5 % in the control group [25], an effect that was maintained 12 months after the intervention period ceased [26]. In Ghana, LNS supplementation has been associated with higher length-for-age z-score when provided to infants from 6–12 months of age compared to a micronutrient tablet or powder [27]. In Burkina Faso, mothers starting LNS in early pregnancy [28] showed an increase in birth length; effects were biggest for those women with low early pregnancy BMI, and in those whose late pregnancy stage occurred during the rainy/lean season [29]. In a separate trial in Burkina Faso, LNS supplementation for children 9–18 mo together with morbidity monitoring and treatment, was associated with a 10 percentage point reduction in stunting [30]. Thus far, few studies have examined developmental outcomes after LNS supplementation and the data available are conflicting. In Malawi, children supplemented from 6–18 months of age with LNS had no difference in age of achievement of motor or language developmental milestones compared to controls [31]. In contrast, in Ghana, the group receiving LNS from 6–12 months of age had a greater proportion of children who could walk independently at 12 months (49 %) compared to a non-intervention group (25 %) [27].

### Question 2: What is the optimal timing and composition of interventions to promote growth and child development?

The existing data and evidence relating to LNS interventions suggest that lipid-based supplements have the potential to improve size at birth and growth through infancy, but the optimal timing and composition of the supplements is not clear. It is possible that if supplementation were to cover *both* mothers and infants during critical periods of growth and development, the effect sizes could be larger than supplementing at one time point alone. Although the evidence from such combined interventions is not yet available, there are some trials of LNS for pregnant women and/or children underway in other settings [32]. Testing the added value of supplementation of pregnant and lactating women over and above supplementation of children during a critical age window of 6–18 months of age will be very informative for understanding the importance of pre- and post-natal nutrition as a strategy for stunting prevention. The biological and economic literature suggests that there are dynamic complementarities between early and later investments in human capital.

### Question 3: What is the most cost-effective way to promote growth and child development and is it scalable within the existing program structure?

Our study will test the cost-effectiveness of LNS targeted to children, as well as pregnant and lactating women, as tools to prevent child stunting and promote optimal child development. While the results from the pregnancy and child LNS supplementation studies are promising, there are important considerations about the cost-effectiveness of these interventions. Given the high prevalence of stunting, and the particularly severe stunting in Madagascar, the rationale for this intervention is strong, particularly if it can be combined with an improved behavior change intervention [33, 34]. The quantities of LNS are intended to be preventative, and thus are less likely to be shared with household members other than the targeted child or pregnant women. Thus, there should be little dilution of the benefits due to food sharing within the household or substitution of existing food intake. While we think that there are likely to be important nutritional benefits of supplementing *both* mothers and their children, it is important to consider that this approach will represent a two year period of supplementation per target child (one year to mothers plus one year of supplementation to their children). Thus, the costs of this intervention will need to be weighed carefully compared to one year of direct supplementation to the child only.

The program under study is already at scale, covering 6500 communities across Madagascar. The community-based program is implemented by the Government and is a key component of the country's nutrition and food security strategy. There are undeniable strengths of the existing program, which builds on an existing large scale infrastructure of service delivery, a strong sense of ownership, and many people intensely dedicated to making the program work. Each of the alternative program designs will compare estimated impacts in terms of reduced stunting and improved cognitive development against the marginal cost of each additional intervention. The preventive new lipid-based supplements are going to be imported from abroad for the purpose of this study. The implementing agency (PNNC/ONN) has already begun discussion for local production of the supplements, however, which would make it a more sustainable approach in the future. The new projected local unit cost of the intervention will be used to assess the benefit/cost ratios of the different packages that include LNS.

### Question 4: What are the synergies and complementarities of integrating nutrition and early stimulation home visits within a large scale program?

There has been a call for large scale programs that integrate health, nutrition and the promotion of child development [12], but there are limited data on the feasibility, advantages, or disadvantages of integrating such interventions. Existing studies consist primarily of small, efficacy trials, and have shown little evidence of synergistic interaction between nutrition and stimulation on child development [35]. There are clear benefits to integrating interventions, because health and nutrition sectors are often the only services reaching children under three years. Thus, delivering support for child development through these mechanisms may be a key way to reach young children, particularly those at risk of poor development. It is also possible that integration can provide additive benefits for children at a lower cost than for a stand-alone program.

### Objectives of the MAHAY study

The objective of the MAHAY (“smart” in Malagasy) study is to determine whether any of the specified intervention packages can significantly alter the pattern of severe growth faltering and delays in child development among young children in Madagascar. The design will not only allow us to measure the impact of each treatment arm compared to the status quo, but also to estimate differential effects and costs of each incremental layer.

Our primary objectives are:

1. Determine whether there are benefits of intensive counseling, nutritional supplementation, or a scaled-up home visiting program for child nutritional status and development, within the structure of an existing government program.
2. Examine the optimal timing and composition of interventions to promote growth and development by comparing intervention arms to each other in terms of effectiveness.
3. Calculate cost-effectiveness of trial arms and explore scalability of various options.
4. Identify where and when synergies and complementarities of integrating early childhood stimulation into the existing nutrition program are feasible.

Our secondary objective is to measure intermediate indicators such as knowledge and hygiene, feeding and stimulation practices that can help us model the pathways through which the different interventions might have an effect on child growth and development.

## Methods/Design

### Overview of the design

The evaluation is a cluster RCT with assignment at the community level. The sample of eligible program communities is assigned to either the current status quo or to one of four arms, based on the randomized design. The study is longitudinal, with sampled children and their households interviewed at three points in time, with a baseline survey administered right before the interventions begin, and two follow-up surveys (midline and endline) administered approximately one and two years after the baseline survey.

Our sampling frame is the universe of eligible project sites in the 5 target regions of the program. From this universe of project communities, a sample of 25 program sites is randomly assigned to one of five groups. The comparison group for our study is the program as currently designed (as opposed to "no program" in most of the evaluation literature). In the four randomized arms, we sequentially add increasing levels of intensity and complexity to the current intervention, starting with the lowest cost option and incrementally adding layers of intensity (and cost) to test the value added of each layer in terms of its ability to reduce growth faltering and promote child development. We will then compare the added value of each layer with its added cost, and will use this information to  assess the cost-effectiveness of alternative modes for service delivery.

In order to identify the variants in program design that could be added to the status quo program, we examined interventions that have shown effectiveness in previous studies. Most of the evidence stems from efficacy trials or small scale interventions, so our objective is to test the implementation within an existing large scale program. Furthermore, we considered interventions that can reasonably be incorporated into the existing program in Madagascar, which is already at scale, will be acceptable to the local population, and that the government would consider adopting as a new package of services.

### Description of the interventions

The interventions are summarized briefly here and then described in more detail below (see also Table 1):

**Table 1:** Summary of intervention components

Study arm	Intensive counseling on nutrition (IC)	IC + LNS for children 6–18 months	IC + LNS for pregnant & lactating women +LNS for children 6-18 months	IC + Early childhood stimulation and development
T_0_) *N* = 25 Communities				
T_1_) *N* = 25 Communities	✓			
T_2_) *N* = 25 Communities	✓	✓		
T_3_) *N* = 25 Communities	✓	✓	✓	
T_4_) *N* = 25 Communities	✓			✓

- T_0_) (comparison) program as currently designed (“status quo”): community-based nutrition program with growth monitoring and education.
- T_1_) Intensive counseling: the existing community-based program enhanced with preventive home visits that focus on personalized counseling.
- T_2_) Intensive counseling + lipid based supplementation to children 6–18 months (“Kalina Zaza”), delivered in a weekly ration of supplement providing 20 g/day/child.
- T_3_) Intensive counseling + lipid based supplementation to children 6–18 months + pregnant and lactating women [−6,+6] (“Kalina Reny”), delivered in a weekly ration of supplement providing 20 g/day/child plus 40 g/day/pregnant women and mothers of children who are 0–6 months old.
- T_4_) Intensive counseling on infant and young child feeding practices + early childhood stimulation for children 6–24 months

#### T_0_: The status quo program

The community-based nutrition program in Madagascar originated from a World Bank financed program (SEECALINE). It is a large-scale program that started in 1999 and was gradually scaled up until 2003 to cover 3600 project sites in more than half of the country’s districts. In 2004/2005 the program was institutionalized and adopted to become the national nutrition program (*Programme National de Nutrition Communautaire* or PNNC), which was subsequently scaled up to cover all districts of the country. To maximize geographical coverage as well as to provide quality services on a large-scale, the program is contracted out to local NGOs for implementation (management, delivery, operations research and supervision) at the local level, reporting to the regional units of the National Nutrition Office. The services are delivered locally by community nutrition workers (*agent communautaire de la nutrition, ACN*), who are usually women elected by the local communities. The basic counseling model was built upon the Essential Nutrition Actions model developed by UNICEF and GAIN [36]. Key messages include information about maternal nutrition, early initiation of breastfeeding, exclusive breastfeeding for the first 6 months, continued breastfeeding through 2 years, dietary diversification, and food conservation and preparation using locally available products, together with the promotion of age-appropriate infant feeding practices and hygiene practices [37]. The messages are delivered at monthly growth monitoring sessions attended by pregnant/lactating women as well all eligible children (0–3 years, subsequently raised to 0–5). The promotion of behavioral change (besides the growth monitoring sessions) includes cooking demonstrations by the community nutrition worker where she emphasizes appropriate complementary feeding practices and prepares recipes that rely on locally available products to promote a healthy and diversified diet.

The long term evaluation (spanning 14 years) showed that during the expansion phase, key aspects of the quality of service delivery (namely the number of children/worker ratios as well the training and the knowledge of community workers) worsened significantly over time [11]. As a consequence, the target age for eligible children was narrowed to 0–2 years, which represents a shift to focus on the first 1000 days.

#### T_1_ Intensive counseling

Given the disappointing long-term results of the existing nutrition support program, the ONN reviewed past experiences, consulted with Alive and Thrive/BRAC in Bangladesh, and introduced an added community nutrition worker (ACDN) fully dedicated to home visits to complement the community worker (ACN) who delivers group growth monitoring activities. The nutrition counseling is designed to reach all children in the community up to 2 years old (one visit during pregnancy, monthly visits during the first 8 months, bimonthly visits during the window of 9–12 months, and quarterly visits from 12 to 24 months). The ACDNs received training with a special emphasis on listening and communication skills, problem solving for exclusive breastfeeding, preventing intestinal worms and promoting sanitation, introduction of complementary feeding, and food security. The counseling interventions are based upon an underlying behavioral theory of change utilizing constructs from an Integrated Behavioral Model (IBM) [38].

#### T_2_ Intensive counseling + LNS for children

In a second arm, 6–18 month old children are supplemented with 20 g/day of LNS, which provide 118 kcal/day and approximately 100 % of the recommended nutrient intakes (RNI) for young children [39]. (Table 2) Families are instructed to mix the 10 g sachets of supplement into their children’s typical food twice per day. A weekly supply of LNS is provided to mothers for all children in the household within the target age of 6–18 months of age by distributing the LNS at the program site by the ACDN. Through the project, LNS distribution logs are utilized to monitor the fidelity of implementation of the LNS delivery.

**Table 2:** Nutrient composition of the maternal and child lipid based nutrient supplements

Nutrient	WHO/FAO RNIs for children 1–3 y^a^	IOM RDAs for pregnant women^b^	LNS child (*Kalina Zaza*)		LNS pregnant/Lactating women (*Kalina Reny*)	
			Content	%RNI	Content	%RDA
Daily dose, g			20		40	
Energy, kcal			118		235	
Fat, g			9.9		19.7	
Protein, g			2.6		5.2	
*Vitamins*						
Vitamin A, μg	400	750	400	100 %	800	107 %
Vitamin D^c^, μg	10	15	10	100 %	15	100 %
Vitamin E, mg	5	15	6	120 %	20	133 %
Vitamin K, μg	15	90	30	200 %	45	50 %
Vitamin C, mg	30	85	30	100 %	100	118 %
Folic acid^d^, μg	150	400	150	100 %	400	100 %
Thiamine (B1), mg	0.5	1.4	0.5	100 %	2.8	200 %
Riboflavin (B2), mg	0.5	1.4	0.5	100 %	2.8	200 %
Niacin, mg	6	18	6	100 %	36	200 %
Pantothenic acid (B5), mg	2	6	2	100 %	7	117 %
Vitamin B6, mg	0.5	1.9	0.5	100 %	3.8	200 %
Vitamin B12, μg	0.9	2.6	0.9	100 %	5.2	200 %
*Minerals*						
Calcium, mg	500	1000	280	56 %	500	50 %
Copper, mg	0.56	1	0.34	61 %	4	400 %
Iodine, μg	90	220	90	100 %	250	114 %
Iron^d^, mg	11.6	60	6	52 %	30	50 %
Magnesium, mg	60	360	40	67 %	150	42 %
Manganese, mg	1.2	11	1.2	100 %	2.6	24 %
Phosphorous, mg	460	700	190	41 %	400	57 %
Potassium, mg	700	4700	200	29 %	1000	21 %
Selenium, μg	17	400	20	118 %	130	33 %
Zinc, mg	8.3	11	8	96 %	30	273 %

^a^RNI for infants and young children 1–3 years. Values from the WHO/FAO Vitamin and Mineral Requirements in Human Nutrition

^b^RDA values for pregnant women from IOM DRIs in 2006

^c^ Vitamin D recommendations are based on IOM RDA for pregnant women and AI for 6–12 months old infants established in 2010

^d^Iron and folic acid recommendations for pregnant women are based on WHO recommendations in areas where anemia prevalence is high

#### T_3_ intensive counseling + LNS for pregnant and lactating women + LNS for children

In this arm, there is an added supplement for pregnant women and breastfeeding women (within the first six months postpartum). The supplement is 40 g/day, providing about 200 kcal/day and 1–2 times the recommended dietary allowance (RDA) of micronutrients for pregnant women (Table 2). A monthly supply of supplements is provided to mothers in a similar way as described above for the child supplements.

For groups T2 and T3, the manufacturer worked with ONN to develop culturally appropriate brand names and packaging for the supplements. In addition, they conducted an acceptability trial including a taste test and two-week, in-home trial to evaluate mothers’ opinions of the product’s taste, consistency, and their ability to integrate it into their usual food preparation practices.

#### T_4_: integrated counseling on nutrition and early stimulation/home visiting

A fourth arm adds home visits for promotion of early stimulation in the home visits. The structured curriculum for early stimulation was adapted from the Jamaica home visiting program [40], as part of a “best practices” collaboration. The materials, books, pictures and training modules were adapted to fit the local context by a team of child development specialists led by the psychologist in the research team [LR]. In order to ensure sufficient intensity of the cognitive stimulation component of the home visit, and considering the workload of the added community nutrition workers (ACDN), only households selected to be participating in the baseline survey were considered eligible to receive the stimulation home visits in the T4 communities. Home visits are bi-weekly, and additional to the home visits for the nutrition counseling, and are tailored to the target child identified in the baseline survey. The curriculum starts at age 6 mo and is administered until the endline survey. Other households with children in T4 target communities that are not sampled for the baseline survey are offered unstructured access to the community site, which is supplemented with a package of toys and books; intervention households also have access to these toys and books.

### Community and participant eligibility criteria, study setting, and enrollment strategy

The five regions included in the study have some of the highest poverty rates in Madagascar and are highly vulnerable to extreme weather shocks (e.g., flooding and drought). The regions are Amoron’i Mania, Androy, Atsimo Atsinanana, Haute Matsiatra, and Vatovavy-Fitovinany. Four of these regions are geographically located in Fianarantsoa province on the south-east coast of Madagascar, and the fifth is located at the very southern tip of the country. Sites eligible for inclusion in the RCT were all active government nutrition program sites in these 5 regions as of January 2014. Sites opened after 2013 are excluded. Each site covers an average of 2–3 small communities of about 1000–2000 inhabitants each, who rely economically on agriculture or fishing, with limited diversification into commerce and trading. Most of the sites have access to a health center and a primary school and many have access to a secondary school. Supervision of government program activities is operated by local Malagasy non-profit organizations (NGOs), which operate uniquely at the regional level.

All pregnant women and women with age-eligible children living in the catchment area of a project site are eligible to participate in the standard growth monitoring and nutritional education that occurs in a group setting in a community center in all sites. The LNS supplement is available to all pregnant/lactating women and age-eligible children in the participating communities assigned to T_3_ or T_2_, respectively. Although no nutritional screening criteria are required for eligibility into the intervention program, severely malnourished children >6 months old will be referred for treatment according to national guidelines. In the T_4_ intervention communities, only women in households selected to be interviewed for the baseline survey are eligible to receive the home visiting and early stimulation component of the program. All other children in the T4 communities who are not part of the baseline survey are eligible for the T_0_ status quo program.

### Outcomes

Primary nutritional outcomes include length/height-for-age and weight-for-length/height (Table 3). Measures of child development are language, cognitive, motor and social development. Secondary indicators include weight-for-age, anemia, and iron status. Intermediate indicators include measures of nutrition and development, and intermediate indicators including dietary diversity, household food security, maternal knowledge of child care and feeding practices, and home stimulation practices. A household survey will be administered with the following questionnaires:

**Table 3:** Outcomes of interest

*Outcome*	*Instrument*	*Notes*	*Timing of data collection*
*Nutritional outcomes:*			
Height/length	Stadiometer (children and caregivers) (locally made, with 1 mm gradations)	Length/height will be measured to the nearest 0.1 cm	Baseline, midline, endline (mothers/caregivers at endline only)
Weight	Electronic mother-baby weighing scale with reading increments of 10 g (SECA Model 874)	Weight will be measured to the nearest 100 g. Duplicate measures will be repeated if the difference between the measurements is >0.1 kg.	Baseline, midline, endline
Anemia/Hemoglobin	Hemocue Hb 301 portable photometer	Measurements will be obtained to the nearest 0.1 g/dL	Baseline, midline, endline (subsample)
Iron and vitamin A status	Enzyme-linked immunoassay (ELISA) method	Capillary blood samples in children 18-24 months	Endline (subsample)
*Child development outcomes:*			
Language and cognitive and socio-emotional development	Ages and Stages Questionnaire: Inventory [51]	Combination of primary caregivers report and direct observation of children 0–36 mo [52]	Baseline, midline, endline
	Bayley Scale [53]	Direct assessment (children 28-34 months)	Endline (subsample)
*Intermediate indicators*			
Household food security	Household Food Security Questionnaire (FANTA)	Questions adapted and modified to reflect the cultural context, as needed.	
Maternal knowledge on nutrition and child development	Questions developed to determine what knowledge is retained from the nutrition education sessions, group activities and/or home visits.	Questions on correct identification of symptoms of illnesses, malnutrition and markers of appropriate child development	Baseline, midline, endline
Food diversity, maternal and child diet	24 h food recall and food diversity score for pregnant women, mothers and children (FANTA, FAO, WHO)	Questions adapted and modified to reflect the cultural context, as needed.	Baseline, midline, endline
Appetite and responsive feeding	Module adapted from Alive and Thrive	Questions adapted and modified to reflect the cultural context, as needed.	Baseline and midline
Maternal child stimulation	Family Care indicator scale (FCI) [54]	Questions to mothers/caregivers regarding household items for child stimulation (e.g. books and toys), and activities (e.g. reading, singing playing).	Baseline, midline, endline
Maternal time use and time spent with child	24 h recall in time spent across different activities, including time spent with target child		Baseline, midline, endline
Maternal receptive vocabulary	Peabody Picture Vocabulary Test (PPVT) [55]	Administered to pregnant women, primary caregivers and community nutrition workers	Baseline
Maternal depressive symptoms	Center for Epidemiologic Studies Depression Scale (CES-D) [56]		Baseline, midline, endline

- a *household* questionnaire with detailed sections on demographics, housing/water and sanitation, education, household expenditures, food security, shocks, and anthropometry. The household questionnaire will be administered to the household head, or in his/her absence to the most informed household member.
- a *female* questionnaire administered to all primary caregivers of the eligible children as well as all pregnant women. Pregnant women will be asked questions on prenatal care, morbidity and a 24 h food recall, knowledge about nutrition, hygiene and stimulation, a depressive symptoms module (based on the Center for Epidemiological Studies-Depression short form, or CESD, but adapted and simplified for use in the survey) and a test of receptive vocabulary (the Peabody Picture Vocabulary Test, or PPVT), which we have used previously in Madagascar [41]. At endline a specific module on LNS will be developed in the LNS treatment arms, with questions about availability, acceptability, and use, as well as perceived benefits from the supplementation.
- a *child* questionnaire administered to all primary caregivers includes a retrospective report on delivery and birth of the child, breastfeeding history and status, timing of introduction of complementary feeding, a module on appetite and responsive feeding, and a 24 h food recall. Mothers or primary caregivers of the target child will also be administered a questionnaire on stimulation practices (using the Family Care Indicators) and a questionnaire on child development (Ages and Stages Questionnaire: Inventory) while this assessment is primarily administered to the mothers/caregivers, there are also opportunities for the children to demonstrate behaviors for the interviewer.
- a *community nutrition worker* questionnaire, with key socioeconomic characteristics, a module on their experience and organization of the program activities, a motivation scale, a knowledge module, and a test of receptive vocabulary (PPVT) [41].
- a *village* questionnaire, with key village infrastructure, village level agro-climatic shocks, and a price questionnaire.

The household survey will be supplemented by *administrative data* coming from the Monitoring and Information System (MIS) of the program, to monitor fidelity and process implementation, as well as key intermediate outcome indicators at the individual and project site level. All household members, together with their children, will be assigned a unique ID that allows optimal integration between survey and MIS data.

### Specialized measurements in subsamples of children at follow-up

(i) **Biomarkers**

The prevalence of anemia, an important risk factor for poor child development, will be measured at baseline, midline and endline using portable Hemocue analyzers. Anemia will be defined as hemoglobin concentration <110 g/L [42, 43]. In serum samples collected at endline, ferritin and transferrin receptor as markers of iron status, retinol binding protein as a marker of vitamin A status, and c-reactive protein (CRP) and alpha-1 acid glycoprotein (AGP) as markers of inflammation will be measured. Iron and vitamin A biomarkers will be adjusted for inflammation using the method described by Thurnham et al. [44].

(ii) **Direct assessment of child development**

The endline survey will include a validation study of the maternal-report ASQ-I instrument to compare it with a direct assessment of child development (the Bayley Scales of Infant Development - BSID). The BSID has been well-validated and provides a strong indication of how children are developing when the test is administered [45]. The BSID requires a trained assessor, and is costly to purchase, which is why we are including it for only a subset of children [46]. The BSID provides scores for both a Mental Development Index and a Motor Development Index, as well as an indicator of socio-emotional development.

### Data quality

Data collection is being carried out by a survey firm with long term experience in collecting health and nutrition data. The team in the survey firm participated in the planning and data collection of regular Demographic Health Survey (DHS) in Madagascar as well as the Anthropometrics and Child Development Surveys (ADE 1997, 2004, 2007, 2011). Through these surveys, the team has developed key expertise in integrating child development tests within a household survey, as well as experience in fielding longitudinal surveys with tracking protocols and data entry in the field [10, 47].

### Data procedures

We collect data with Computer Assisted Personalized Interviews (CAPI) using Android tablets, using a free platform developed by the World Bank (solutions.worldbank.org). All documentation regarding the participants, including the laboratory samples, source data and the questionnaires, will be identified using appropriate participant codes. All records will be kept in a secure server and will be accessible only to approved survey firm personnel and research team members. Clinical information will not be released without written permission of the subject, except as necessary for clinical monitoring. Data from these registers may be given only to authorized members of the research team and authorities guiding health research in Madagascar.

In addition, we collect monthly administrative data on all the treatment arms for the added community nutrition worker, in addition to the regular weighing data from the growth monitoring activities. In addition, midline and endline survey data will have a participation module with a detailed account of participation in all program activities.

### Costing data

Unit costs for the “status quo” program are available from existing project data and are approximately 15 USD per target beneficiary. The total cost includes the wages of the community worker, the training cost (refresher) of the community worker, the wages, training and supervision cost (NGO and district supervisor), the unit cost of the cooking demonstration, the information, education and communication (IEC) materials, the equipment and registries at the community level, and the health card of the child.

The additional cost of the intervention included in the intensive counseling arm (T1) will add additional salary, training and supervision of the added community worker.

For the purpose of this study, the LNS supplements are produced internationally and imported from overseas, which results in a substantial addition to the unit cost in the supplementation arms. The actual costing for the cost-effectiveness analysis (and for informing scale up of the study) will include the scenario of local production around which the ONN has already begun discussions.

During the implementation of this study, districts and NGO supervisors across all intervention arms will be provided with costing data sheets to record actual expenditures incurred at regular points during implementation. A framework is being developed to monitor these costs over time, which will feed into the cost benefit analysis work.

Last, we plan to collect information to incorporate private costs to mothers. We include a small time use section to be administered to the primary caregiver, with questions related to the time needed to participate in each program activity. We will also have wage rates for agriculture and casual labor in the community questionnaire, which are likely to represent an upper bound on the opportunity cost of time. The information will be triangulated with the history of seasonal wage rates available from the commune census collected by Cornell and Oxford University (http://www.ilo.cornell.edu/ilo/data.html). The opportunity costs will be highly dependent on the season, so we want to make sure that the full accounting of private costs will not be a function of the time of the interviews.

### Referral guidelines

The study will refer participants for treatment if we observe either of the two following outcomes: soy or nut allergies that would make it potentially dangerous to consume LNS supplementation, and/or acute malnutrition.

#### Soy or nut allergies related to LNS

In the LNS arms, health promoters will recommend that caregivers stop using the LNS should their child have any adverse reactions shortly after ingesting the supplement (such as vomiting, stomach pain, rash, breathing problems with wheezing). In the event of an adverse reaction, the health promoters will refer the child, if necessary, to the closest health facility for treatment.

#### Acute malnutrition

In the assessment survey, children who are found to be severely malnourished based on WHO/Unicef criteria (severely wasted [WHZ < −3] and/or bipedal edema) will be referred to health facilities with intensive nutrition rehabilitation centers for treatment.

### Sample size

The number of communities was selected based on testing the null hypothesis that the treatment effect for any pair-wise comparison of treatment arms is equal to zero at a conventional 95 % confidence level (alpha = 0.05) to achieve a statistical power of 80 %.

An effect size of 0.25 to 0.30 SD for the supplementation arms is expected to be seen based on the review on lipid-based complementary feeding interventions in developing countries [14] and was chosen for the mid-point of the sample size calculation.

The intra-cluster correlation was drawn from the 2011 Anthropometrics Survey for height-for-age z-scores (HAZ). With an effect size of 0.30 SD and an estimated intra-cluster correlation of 0.10, the number of communities to be included in each pair-wise comparison of treatment arms should be 50 communities (25 per treatment arm) with 30 children sampled from within each community.

### Randomization and blinding

#### Sampling of 125 Sites

Site selection and randomization to intervention arm was carried out by the research team in Washington DC using January 2014 governmental administrative data of active program sites, which indicated the existence of 2000 operating sites in the five targeted regions. Newly opened sites were excluded from the randomization process, reducing the sampling frame of eligible study sites to 1476.

Because of regional differences in prevalence of malnutrition, food security, and income opportunities, the sample was stratified at the regional level. In addition, there are 89 NGOs in total in the five targeted regions. Each NGOs has on average five supervisors (median = 3) and each supervisor oversees on average eight sites.

Sampling of study sites was performed in three stages using a statistical software program to generate uniformly distributed random numbers for the sites. First, to minimize contamination by NGO supervisor, we randomly sampled one site per supervisor, such that each person supervised at most one intervention type, reducing the number of sites to 261. Second, in order to reduce the logistical constraints of working with a large number of NGOs, we randomly sampled 13 NGOs per region, for a total of 65 NGOs. Finally, in the third stage, we assigned sites to a trial arm by performing a stratified random sample of 5 sites per intervention arm per region for a total of 125 sites (25 sites per region). The final sample was balanced across trial arms along average underweight prevalence and average size of the target population, based on program monitoring data. Local Malagasy government officials were notified of the site selection and program assignment after the above process was completed by the research team.

The compliance with the community random assignment is closely monitored by the implementing agency in collaboration with the research team.

#### Sampling of children

The initial sampling frame of survey participants within selected community sites was based on a census performed by the local community nutrition workers in November/December 2013. These same nutrition workers provided updates to the census in April 2014 for all sites and up to a week before the team’s arrival for each individual site. Upon the team’s arrival for the baseline data collection, the list received a final update with the help of the nutrition worker to add newborns and newly pregnant women, update that status of pregnant women who had given birth, and remove those who were traveling, had migrated or died. In the case where we had an insufficient number of pregnant women or children in the updated list, then the catchment area was extended to villages within 5 km of the site that do not currently participate in the government program, but are eligible to participate.

The final sample is stratified in the following way: pregnant women, women with children 0–5 months of age, and women with children 6–11 months of age. Ten eligible participants were systematically sampled from each stratum using a random starting point and sampling interval. In each stratum, we over-sampled by two additional women to anticipate needing to exclude households from the sample for three possible reasons. The first reason women were over-sampled was to be prepared for possible refusals. Second, in order to avoid clustering within households, if two women were found to be from the same household, one was replaced (so that no household was represented twice). Pregnant women were given priority, followed by the woman with the youngest child in the household. Finally, screening questions were used during the household interview to exclude children with a serious developmental disability (sight, hearing, physical, or mental). The final sample of 30 women and their respective households are interviewed at baseline, midline and endline. A time difference of at least 18 months allows for sufficient exposure to the program and account for potential delay in implementation of the intervention.

All children at midline and endline will be tested for anemia as measured by hemoglobin concentration, a secondary outcome measure. Additionally, a subsample of 384 children at endline will be directly assessed for biomarker data and direct assessment of their cognitive, language, motor and socioemotional development using the BSID. The specialized data collection will take place in a random sample of 64 communities stratified by treatment status. In each of these 64 communities, 6 children aged 18-24 months of age will be administered the biomarker data (ELISA method) and 6 children aged 28-34 will be administered the BSID.

#### Blinding

Due to the nature of the interventions, it is impossible to blind participants to their intervention group assignment. Interviewers will not be blinded either as they will ask participants and community health workers about their program-related experiences. However, the data will be masked to group assignment during the data analysis phase to blind the data analysts.

### Analysis plan

#### General analytic approach

Primary analyses will be performed on an intention-to-treat (ITT) basis, to minimize the selection bias that could occur if there are selective take-up rates. The intention-to-treat provides the lower bound on the estimated impact of the program, and represents the parameter of interest for cost-benefit analysis [48].

Identification of causal impacts will rely on the random assignment of treatment at the community level. The main impact of the program activities and alternative treatment arms will be estimated through a series of pair-wise comparisons of mean outcomes between the study arms. However, due to the small number of communities assigned to each arm (*n* = 25), it is possible that by chance alone we will find covariate imbalance between communities in different treatment arms. Therefore, we will measure important covariates at the individual and community level in order to include them as a subset of conditioning variables in a regression framework.

The potential for contamination or spillover effects across communities for the program currently implemented is low, as community sites are self-contained and provide services to mothers and children registered in the local community. The concern, however, may arise for communities that distribute LNS for free. Monitoring distribution to eligible mothers and children through the administrative data from the program monitoring system, as well as sampling communities with a sufficient buffer zone/minimum distance among them, should minimize the extent of contamination.

There are no plans for broad-scale social marketing of LNS outside of information materials developed for the purposes of this study. LNS is not available for purchase or for free through government or any other NGO programs. The only way to obtain it will be through this project. For these reasons, we anticipate little contamination of control areas. We will continue to monitor the availability of LNS in non-study areas throughout the course of the trial to determine if this situation should change in the future. We acknowledge the possibility of sharing with other household members or other community members. To minimize this risk, all individuals in the community in the designated age range or pregnancy/postpartum window will be eligible to receive LNS, regardless of whether or not they participate in the evaluation. Communication material and instructions provided to mothers will discourage sharing and explain how the LNS has been developed specifically for children 6–24 months or pregnant/lactating women.

#### Parameters of interest

In this section, we introduce notation to simplify the description of our parameters of interest. Treatment, D assigned at the community level, is represented by four arms: Intensive counseling (I), Intensive counseling + Nutrition for children 6–18 months (IN_C_), Intensive counseling + Nutrition for pregnant/lactating mothers and children 6–18 mo (IN_MC_), and Intensive counseling + child Stimulation (IS). The status quo, or control arm, is denoted by C. In notation terms, the full set of potential assignment variants is given by *t* = [C, I, IN_C_, IN_MC_, IS]. All our specifications will control for the method of randomization by including strata dummies, and correcting standard errors for clustering at the site level. A set of individual and community level covariates are represented by X, and will be used to adjust for imbalance that may have occurred by chance in the randomization process.

**Question 1 : Estimands Ψ**^**I-IV**^: d = [I, IN_C_, IN_MC_, IS] vs. C

The estimands are four pair-wise comparisons of an enhanced intervention arm with the control arm, and are represented by the following equation:

Ψ^*I* − *IV*^ = *E*(*E*(*Y*|*D* = *d*, *X*) − *E*(*Y*|*C*, *X*)), where *d* = [I, IN_C_, IN_MC_, IS].

**Question 2: Estimand Ψ**^**V**^: IN_MC_ vs. IN_C_

The fifth pair-wise comparison is between the two supplementation arms:

$$ {\Psi}^V=E\left(E\left(Y\Big|I{N}_{MC},X\right)-E\left(Y\Big|I{N}_C,X\right)\right) $$

**Question 3: Estimands Ψ**^**VIII-X**^: *d* = [IN_C_, IN_MC_, IS] vs. I

These final estimands are three pair-wise comparisons of the intensive counseling plus supplementation arms or the intensive counseling plus stimulation arm with the intensive counseling alone arm. The estimands are represented by:

Ψ^*VIII* − *X*^ = *E*(*E*(*Y*|*D* = *d*, *X*) − *E*(*Y*|*I*, *X*)), where *d* set [IN_C_, IN_MC_, IS]

#### Testing and estimation

We will use unadjusted linear regression for the initial estimation of our causal parameters for the linear outcome measures (e.g., anthropometric z-scores, developmental outcomes) and unadjusted logistic regression for binary outcomes, such as stunting, severe stunting, wasting, severe wasting, and anemia. We will complement these estimations using parametric regression, adjusting for key covariates, X, that may be associated with treatment assignment by chance alone, as well as adjusting for baseline differences in the outcomes of interest. Our extended parametric specification will be the following:

$$ {Y}_{ijt}={\beta}_0+\sum_{d=1}^4{\beta}_{1d}\left({D}_j=d\right)+{\gamma}_0{Y}_{ij0}+{\Gamma}_s^{{\textstyle \hbox{'}}}\theta +{\lambda}_t+{\varepsilon}_{ijt} $$

where *Y*_*ijt*_ is the outcome of interest for individual, i, and community, j at post-treatment at time *t*, *Y*_*ij*0_ is its baseline value, 1(*D*_*j*_ = *d*) is an indicator for the treatment, *d*, assigned to a community, Γ_*s*_^'^ is a vector of randomization strata dummy variables (region and age group), and *λ*_*t*_ is a dummy indicator for the post-treatment rounds (midline vs endline). The coefficient *β*_1__*d*_ provides the intention to treat effect, which is the effect of being in a village that was offered the program program variant *d*.

Standard errors for the parametric estimates will be adjusted for village level clustering. We will adjust for multiple hypothesis testing by grouping our outcome measures into domains, using standardized treatment effects within that domain and adjusting for multiple inference [49]. Missing data on the outcomes at baseline will be dummied out. With detailed tracking protocols in place, we do not expect significant attrition at one-year intervals between baseline, midline and endline. To the extent that attrition is observed and is correlated with treatment status, we will obtain bounds on the effects using Lee bounds [50].

#### Estimation of heterogeneity treatment effects

Heterogeneous treatment effects will be estimated by interacting the treatment status (and all control variables) with the variable of interest. We will look at interaction effects along the following key dimensions: (i) strata (age and region) (ii) pre-program child outcomes (such as anthropometric z-scores, anemia and ASQ-I scores), (iii) pre-program characteristics of the primary caregiver (such as education attainment, receptive vocabulary and height, depressive symptoms at baseline) or her household (such as access to safe water, food security and wealth indicators at baseline), and pre-program village characteristics (such as remoteness/accessibility and average food security).

#### Midline data analysis

Preliminary intervention effect estimates will be obtained following the one-year midline study in order to assess potential near-term protective effects of the program during periods of severe weather shocks. However, decisions to stop or change the intervention protocols will not be made solely based on these estimates for two main reasons. First, a 12-month follow-up period is an insufficient amount of time for the youngest children to have received a full course of treatment in the LNS and stimulation arms. Second, using data from all three time points will give a more complete and reliable understanding of the impacts of the different treatment arms in our study, and how the relative effectiveness across the arms may vary over time.

### Stopping guidelines

No external data safety monitoring board is pre-planned, but quality control during data collection is ensured by the Madagascar Office of National Nutrition using their standard operating procedures and regular collection of the monitoring data system. We do not anticipate serious risk to our participants from participating in the study. However, there may be adverse consequences to participation that were unintended or unexpected. In the absence of a local or external monitoring board, we rely on local internal monitoring for reports of adverse events, as well as preliminary analyses following the midline study, to stop any intervention arm if clear evidence of harm is found from program participation. In the event that the monitoring reports or midline analyses indicate evidence of substantial early benefit from one of the intervention arms, we will continue the trial, with the Government’s approval, in order to capture differential effects of the intervention arms over time and by age cohort of the children.
